# Supplementary material for: Unveiling the Bioactive Potential of the Invasive Jellyfish Phyllorhiza punctata Through Integrative Transcriptomic and Proteomic Analyses
Source: Biomolecules. 2025 Aug 4;15(8):1121. doi: 10.3390/biom15081121 (PMC12383608; doi:10.3390/biom15081121)
Supplement: Supplementary file 1 [file biomolecules-15-01121-s001.zip › Suplementary Material/FileS3/PEPFOLD4/XP_065060867_704/clusters.html]

 PEP-FOLD clusters


Download archive of all models  here   

|  |  |  |
| --- | --- | --- |
| # | file | sOPEP |
| # cluster 1 - Size: 10 |  |  |
|  | XP\_065060867\_704-model1 | -18.2939 |
|  | XP\_065060867\_704-model1.1 | -18.2283 |
|  | XP\_065060867\_704-model1.2 | -18.2283 |
|  | XP\_065060867\_704-model1.3 | -18.127 |
|  | XP\_065060867\_704-model1.4 | -18.127 |
|  | XP\_065060867\_704-model1.5 | -18.127 |
|  | XP\_065060867\_704-model1.6 | -18.127 |
|  | XP\_065060867\_704-model1.7 | -18.0746 |
|  | XP\_065060867\_704-model1.8 | -18.0746 |
|  | XP\_065060867\_704-model1.9 | -18.0582 |
| # cluster 2 - Size: 8 |  |  |
|  | XP\_065060867\_704-model2 | -18.2786 |
|  | XP\_065060867\_704-model2.1 | -18.0842 |
|  | XP\_065060867\_704-model2.2 | -18.0842 |
|  | XP\_065060867\_704-model2.3 | -18.0743 |
|  | XP\_065060867\_704-model2.4 | -18.0687 |
|  | XP\_065060867\_704-model2.5 | -18.0453 |
|  | XP\_065060867\_704-model2.6 | -18.0453 |
|  | XP\_065060867\_704-model2.7 | -18.0453 |
| # cluster 3 - Size: 8 |  |  |
|  | XP\_065060867\_704-model3 | -18.2542 |
|  | XP\_065060867\_704-model3.1 | -18.2258 |
|  | XP\_065060867\_704-model3.2 | -18.2258 |
|  | XP\_065060867\_704-model3.3 | -18.2258 |
|  | XP\_065060867\_704-model3.4 | -18.1772 |
|  | XP\_065060867\_704-model3.5 | -18.1251 |
|  | XP\_065060867\_704-model3.6 | -18.0849 |
|  | XP\_065060867\_704-model3.7 | -18.0849 |
| # cluster 4 - Size: 4 |  |  |
|  | XP\_065060867\_704-model4 | -18.2318 |
|  | XP\_065060867\_704-model4.1 | -17.9945 |
|  | XP\_065060867\_704-model4.2 | -17.9925 |
|  | XP\_065060867\_704-model4.3 | -17.9778 |
| # cluster 5 - Size: 9 |  |  |
|  | XP\_065060867\_704-model5 | -18.2258 |
|  | XP\_065060867\_704-model5.1 | -18.2258 |
|  | XP\_065060867\_704-model5.2 | -18.1274 |
|  | XP\_065060867\_704-model5.3 | -18.1274 |
|  | XP\_065060867\_704-model5.4 | -18.1274 |
|  | XP\_065060867\_704-model5.5 | -18.1274 |
|  | XP\_065060867\_704-model5.6 | -18.1274 |
|  | XP\_065060867\_704-model5.7 | -18.1274 |
|  | XP\_065060867\_704-model5.8 | -18.1274 |
| # cluster 6 - Size: 1 |  |  |
|  | XP\_065060867\_704-model6 | -18.2003 |
| # cluster 7 - Size: 6 |  |  |
|  | XP\_065060867\_704-model7 | -18.1588 |
|  | XP\_065060867\_704-model7.1 | -18.1588 |
|  | XP\_065060867\_704-model7.2 | -18.1588 |
|  | XP\_065060867\_704-model7.3 | -18.1588 |
|  | XP\_065060867\_704-model7.4 | -18.1588 |
|  | XP\_065060867\_704-model7.5 | -18.1588 |
| # cluster 8 - Size: 13 |  |  |
|  | XP\_065060867\_704-model8 | -18.1544 |
|  | XP\_065060867\_704-model8.1 | -18.1544 |
|  | XP\_065060867\_704-model8.2 | -18.1544 |
|  | XP\_065060867\_704-model8.3 | -18.1544 |
|  | XP\_065060867\_704-model8.4 | -18.1252 |
|  | XP\_065060867\_704-model8.5 | -18.1252 |
|  | XP\_065060867\_704-model8.6 | -18.1252 |
|  | XP\_065060867\_704-model8.7 | -18.1252 |
|  | XP\_065060867\_704-model8.8 | -18.1252 |
|  | XP\_065060867\_704-model8.9 | -18.1252 |
|  | XP\_065060867\_704-model8.10 | -18.1252 |
|  | XP\_065060867\_704-model8.11 | -18.1064 |
|  | XP\_065060867\_704-model8.12 | -18.1064 |
| # cluster 9 - Size: 3 |  |  |
|  | XP\_065060867\_704-model9 | -18.1533 |
|  | XP\_065060867\_704-model9.1 | -18.1533 |
|  | XP\_065060867\_704-model9.2 | -18.1533 |
| # cluster 10 - Size: 2 |  |  |
|  | XP\_065060867\_704-model10 | -18.1467 |
|  | XP\_065060867\_704-model10.1 | -18.0207 |
| # cluster 11 - Size: 3 |  |  |
|  | XP\_065060867\_704-model11 | -18.118 |
|  | XP\_065060867\_704-model11.1 | -18.118 |
|  | XP\_065060867\_704-model11.2 | -18.0168 |
| # cluster 12 - Size: 2 |  |  |
|  | XP\_065060867\_704-model12 | -18.1165 |
|  | XP\_065060867\_704-model12.1 | -18.1165 |
| # cluster 13 - Size: 2 |  |  |
|  | XP\_065060867\_704-model13 | -18.1148 |
|  | XP\_065060867\_704-model13.1 | -18.0467 |
| # cluster 14 - Size: 4 |  |  |
|  | XP\_065060867\_704-model14 | -18.1049 |
|  | XP\_065060867\_704-model14.1 | -18.0678 |
|  | XP\_065060867\_704-model14.2 | -18.0678 |
|  | XP\_065060867\_704-model14.3 | -18.0285 |
| # cluster 15 - Size: 7 |  |  |
|  | XP\_065060867\_704-model15 | -18.0905 |
|  | XP\_065060867\_704-model15.1 | -18.0905 |
|  | XP\_065060867\_704-model15.2 | -18.0905 |
|  | XP\_065060867\_704-model15.3 | -18.0905 |
|  | XP\_065060867\_704-model15.4 | -18.0905 |
|  | XP\_065060867\_704-model15.5 | -18.0905 |
|  | XP\_065060867\_704-model15.6 | -18.0617 |
| # cluster 16 - Size: 5 |  |  |
|  | XP\_065060867\_704-model16 | -18.0898 |
|  | XP\_065060867\_704-model16.1 | -18.0898 |
|  | XP\_065060867\_704-model16.2 | -18.0628 |
|  | XP\_065060867\_704-model16.3 | -18.0628 |
|  | XP\_065060867\_704-model16.4 | -17.9896 |
| # cluster 17 - Size: 2 |  |  |
|  | XP\_065060867\_704-model17 | -18.0874 |
|  | XP\_065060867\_704-model17.1 | -18.0874 |
| # cluster 18 - Size: 1 |  |  |
|  | XP\_065060867\_704-model18 | -18.0639 |
| # cluster 19 - Size: 6 |  |  |
|  | XP\_065060867\_704-model19 | -18.062 |
|  | XP\_065060867\_704-model19.1 | -18.062 |
|  | XP\_065060867\_704-model19.2 | -18.062 |
|  | XP\_065060867\_704-model19.3 | -17.997 |
|  | XP\_065060867\_704-model19.4 | -17.997 |
|  | XP\_065060867\_704-model19.5 | -17.9853 |
| # cluster 20 - Size: 1 |  |  |
|  | XP\_065060867\_704-model20 | -18.0302 |
| # cluster 21 - Size: 3 |  |  |
|  | XP\_065060867\_704-model21 | -18.0222 |
|  | XP\_065060867\_704-model21.1 | -18.0222 |
|  | XP\_065060867\_704-model21.2 | -18.0222 |
|  |  |  |
